# Streamlining neuroradiology workflow with AI for improved cerebrovascular structure monitoring

**DOI:** 10.1038/s41598-024-59529-y

**Published:** 2024-04-22

**Authors:** Subhashis Banerjee, Fredrik Nysjö, Dimitrios Toumpanakis, Ashis Kumar Dhara, Johan Wikström, Robin Strand

**Affiliations:** 1 Department of Information Technology, Uppsala University, Uppsala, Sweden; 2 Department of Surgical Sciences, Neuroradiology, Uppsala University, Uppsala, Sweden; 3 Department of Electrical Engineering, National Institute of Technology Durgapur, Durgapur, India

**Keywords:** Biomedical engineering, Cerebrovascular disorders, Brain imaging, Magnetic resonance imaging

## Abstract

Radiological imaging to examine intracranial blood vessels is critical for preoperative planning and postoperative follow-up. Automated segmentation of cerebrovascular anatomy from Time-Of-Flight Magnetic Resonance Angiography (TOF-MRA) can provide radiologists with a more detailed and precise view of these vessels. This paper introduces a domain generalized artificial intelligence (AI) solution for volumetric monitoring of cerebrovascular structures from multi-center MRAs. Our approach utilizes a multi-task deep convolutional neural network (CNN) with a topology-aware loss function to learn voxel-wise segmentation of the cerebrovascular tree. We use Decorrelation Loss to achieve domain regularization for the encoder network and auxiliary tasks to provide additional regularization and enable the encoder to learn higher-level intermediate representations for improved performance. We compare our method to six state-of-the-art 3D vessel segmentation methods using retrospective TOF-MRA datasets from multiple private and public data sources scanned at six hospitals, with and without vascular pathologies. The proposed model achieved the best scores in all the qualitative performance measures. Furthermore, we have developed an AI-assisted Graphical User Interface (GUI) based on our research to assist radiologists in their daily work and establish a more efficient work process that saves time.

## Introduction

The use of radiological imaging is critical in the diagnosis and comprehension of various vascular diseases and neurological disorders that affect the intracranial cerebrovascular structure, including intracranial aneurysms, arteriosclerosis, and arteriovenous malformations. Additionally, such imaging is crucial for post-operative follow-up and pre-operative planning. Noninvasive medical imaging techniques, such as Time-Of-Flight Magnetic Resonance Angiography (TOF-MRA)^1^, are frequently used to obtain image data of blood vessels without the use of contrast agents. Inspection of the intracranial cerebrovascular structure with 3D TOF-MRA images is most commonly performed either by manual slice-by-slice inspection of the 3D volume image or by Maximum Intensity Projection (MIP)^2^. However, manual volume measurement of vascular anomalies related to pre and post-operative planning and evaluation is a tedious task that is subject to interobserver variability, human error, and bias. The cerebrovascular structure from TOF-MRA can be visualized by 3D renderings of computerized segmentation of the structure. This is an appealing alternative for facilitating time-critical diagnosis of vessel abnormalities. Inter-subject variation in cerebrovascular topology and complex geometry, and data-specific hazards like noise, sparsity, and artifacts make computerized segmentation extremely difficult^3^.

Various vessel segmentation methods have been proposed in the literature, including model-driven or classical image analysis-based techniques such as simple thresholding-based, region growing, multiscale vesselness filtering, and statistical shape modeling. However, these models face difficulties in producing acceptable segmentations, often requiring feature engineering and manual parameter selection. To overcome these limitations, recent studies have shown that Deep Convolutional Neural Networks (CNNs) can achieve better vessel segmentation accuracy by utilizing contextual information and high-level feature extraction capabilities^4,5^. Encoder-decoder structures based on CNNs have gained attention for 2D and 3D segmentation of the cerebrovascular structure from TOF-MRA images. Uception^6^, DeepVesselNet^7^, BRAVE-NET^4^, JointVesselNet^8^, and VC-Net^9^ are examples of such networks proposed for 3D volumetric cerebrovascular segmentation from TOF-MRA images. Uception uses Inception modules within the U-Net structure, while DeepVessel-Net approximates the effect of 3D kernels in multiple orthogonal planes by using 2-D cross-hair filters to reduce memory and computational complexity. BRAVE-NET incorporates deep supervision and context aggregation within the baseline U-Net^10^ architecture to preserve small vessel structures. JointVesselNet and VC-Net are similar networks that propose using both 2D and 3D U-Nets, which are jointly trained on 3D volumetric patches and 2D MIP patches of the corresponding 3D patch.

**Figure 1:**
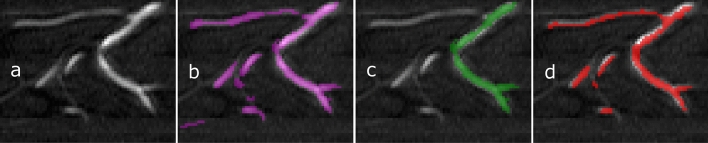
(**a**) Shows TOF-MRA patch (the vessels are shown as hyperintensities). (**b**) shows manual segmentation (in magenta). In (**c**,**d**), segmentations generated from two deep learning-based models are shown in green (Dice score 0.672) and red (Dice score 0.674).

Deep CNN-based segmentation methods have made significant progress, but they still face challenges in accurately segmenting curvilinear and tubular structures such as vascular structures. In Fig. 1, the red segmentation shows good performance in capturing the topological structure, although it is not perfect^11^. On the other hand, green segmentation performs well in segmenting large vessels, but it struggles with small and thin vessels. Given the preference for topology, connectivity, and structures, red segmentation is preferred. However, the traditional Dice score is not a reliable quality measure for curvilinear and tubular structure segmentation since it evaluates similar values for both segmentation results (0.67). Despite this, it has become a common practice in the literature to use the Dice score as the loss function to train deep segmentation models. This practice can induce a strong bias towards accurately segmenting large vessels rather than preserving global network connectivity, leading to suboptimal results.

**Figure 2:**
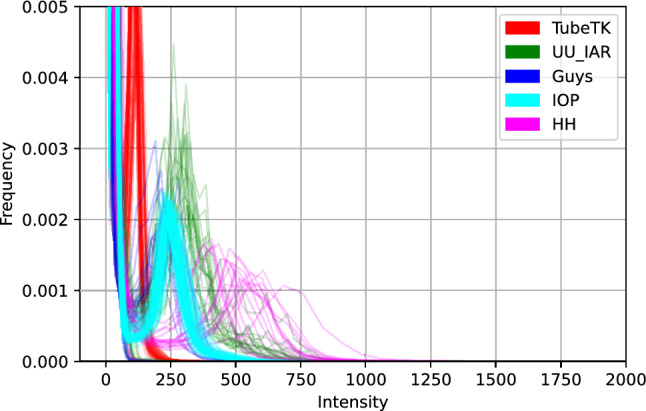
The overall intensity histogram distributions of the MRA images from five sites.

Along with the aforementioned issue, it has been shown that data-driven approaches fail to generalize well when applied to multi-center datasets. MRA images coming from different centers have inter-scanner variability (Fig. 2), which affects the downstream voxel-based analysis. Combining multi-center imaging data is challenging as there is no standardization in image acquisition protocols, software, and scanner hardware (scanner drift, scanner upgrade, scanner strength, etc.). Another important concern is variability in the sample demographics, which should be carefully managed when combining data from multi-sites. Due to such issues, a large difference between training and test data (coming from different centers) is observed and often termed “domain shift”^12^. Several methods have been proposed in the literature to tackle the issue^12–14^. We can broadly divide it into two groups viz. using massive data preprocessing or improving models’ generalization capacity through improved training strategies to handle domain shifts. Data preprocessing-based techniques use multiple sequential steps to map the multi-center neuroimaging datasets into a common reference space. It typically starts by selecting a reference image or an atlas image and normalizing the intensities of all the other images using some linear histogram matching method as proposed by Nyul et al.^15^. Finally, the images are spatially normalized into a common isotopic atlas reference space such as Montreal Neurological Institute (MNI) reference space. Denoising, bias field correction, etc. are sometimes also performed before registration. Although this is the most commonly used technique in practice it is very time-consuming and needs a manual selection of parameters as well as reference images, which makes it unsuited for real application scenarios. In the recent literature, it is referred to as MRI harmonization, and methods such as Unlearning dataset bias for multi-center MRI have been addressed through network training strategies.

One of the most popular methods to achieve it is DANN (Domain Adversarial training of Neural Networks)^16^ which uses a gradient reversal layer to adversarially learn domain information to maximize performance on the main task while removing domain information. Inspired by DANN Dinsdale et al.^17^ proposed a deep learning-based training scheme that creates scanner-invariant features for multi-site MRI using an iterative update approach. For diffusion MRI, Moyer et al.^18^ use variational autoencoders to create scanner-invariant representations of the data. The generalized representations may then be used to recreate the input images so that they lose the correlation with the original collection site. Generative models, mostly based on deep learning such as Encoder-Decoder networks^10^, GANs^19,20^, variational autoencoders^18^ have been employed to harmonize multi-site MRI data. Heuristic techniques and randomization methods such as early stopping^21^, weight decay^22^, dropout^23^, and data augmentation^24^ is also used for improving the models’ generalization. The domain adaptation-based approaches are limited by the fact that it requires iterative adversarial training and can not be achieved in a single step. In the case of Generative methods, generated “harmonized” images are hard to validate and require the active participation of experienced radiologists. Risks of unknown errors propagating through pipelines have the potential to alter the results of any completed analysis.

This paper addresses the aforementioned issues by presenting and evaluating a topology-aware learning strategy with a Decorrelation Loss (DcL) for volumetric cerebrovascular segmentation from multi-center MRAs. The topology-guided learning involves training a multi-task deep CNN along with a topology-aware loss function proposed in Ref.^3^. While clDice^25^ is also proposed for ensuring topological consistency, it relies on min- and max-pooling, which we found unsuitable for thick vessel structures, such as cerebral vessels, and specifically, the circle of Willis. In cases involving MRA data, this approach leads to the generation of erroneous and discontinuous vessel centerlines. The primary task in the multi-task deep CNN focuses on learning voxel-wise segmentation of the cerebrovascular tree in parallel with two sub or auxiliary tasks. The auxiliary tasks are to (i) learn the distance from the voxels on the surface of the vascular tree by utilizing a distance transform and (ii) learn the vessel centerline. Recent literature^26^ has shown that training a multi-task model with sub or auxiliary tasks boosts the performance of the main task. In practice, this approach provides additional regularization and allows the encoder to learn more high-level intermediate representations. To diminish the effect of domain differences in the multi-center MRAs the encoder network of the proposed model is aimed to learn generalized features that the decoder network will use further. We propose to achieve this using a regularization network at the end of the encoder network, which acts as a domain-regularization for the encoder network. The advantage of the proposed approach is that it does not require an iterative adversarial training phase and can learn generalized features during the main training phase only.

The primary goal of this paper is to propose an end-to-end AI-based solution for enhanced monitoring of cerebrovascular structures. To achieve this, we addressed various aspects, including handling domain shifts in multi-center data and utilizing a loss function for better preservation of topology^3^. Additionally, we developed a Graphical User Interface (GUI) that supports visualization and interactive annotation to assist radiologists in their daily work and establish a time-saving workflow. The GUI was implemented in Python and OpenGL within a zero-footprint application environment. This GUI can generate a 3D reconstruction of the cerebrovascular tree from an input 3D MRA scan, providing tools for semi-automated quantification of vascular pathologies from the MRA volume. Through experimental studies, we demonstrated that artificial intelligence (AI) technology can be seamlessly integrated into the clinical workflow to enhance efficiency and reduce medical costs. In addition to these contributions, we conducted rigorous testing, validation, and comparisons with state-of-the-art methods, both quantitatively and qualitatively. Our analysis also extended to evaluating the performance of the developed methods in terms of multi-center dataset generalization and pathology-preserving vessel segmentation.

## Experiments and results

### Dataset

Retrospective data with and without vascular pathologies were collected from multiple private and public data sources scanned at six different hospitals. We analyzed four publicly available datasets viz. “ITKTubeTK” (from CASILab, University of North Carolina at Chapel Hill (https://public.kitware.com/Wiki/TubeTK/Data), “HH” (from Hammersmith Hospital, Imperial College London), “Guys” (from Guy’s Hospital, London), and “IOP” (Institute of Psychiatry, King’s College London) contains TOF-MRA images of the brain from healthy subjects. We used another cohort of patients with at least one diagnosed Unruptured Intracranial Aneurysm (UIA) and cohorts of persons screened for UIAs because of a positive family history for aneurysms Subarachnoid Haemorrhage (aSAH) scanned at the University Medical Center (UMC), Utrecht. This brain TOF-MRA dataset was released by the “Aneurysm Detection And segMentation (ADAM)” Challenge organized in conjunction with MICCAI 2021. One in-house clinical TOF-MRA image dataset (prospective research project, approved by the local ethical committee) of Intracranial Aneurysm Remnant (IAR) named “UU-IAR” was collected from the Uppsala University hospital. Endovascular intervention was performed to remove a large portion of the aneurysm. Parameters of the TOF imaging of each dataset are summarized in Table 1.

**Table 1:** Detailed description of different datasets, scanning protocols, and number of CT images from different manufacturers.

Dataset	Voxel size ($mm^3$)	Matrix size	TR/TE (ms)	Flip angle (degrees)	Scanner	# of images
ITKTubeTK	$0.5\times 0.5\times 0.8$	$448\times 448\times 128$	35/3.56	22	Siemens 3T	109
HH	$0.5\times 0.5\times 0.8$	$512\times 512\times 100$	16.72/5.75	16	Philips 3T	181
IOP	$0.3\times 0.3\times 0.8$	$1024\times 1024\times 92$	26/4.2	25	GE 1.5T	73
Guys	$0.5\times 0.5\times 0.8$	$512\times 512\times 100$	20/6.91	25	Philips 1.5T	316
UU-IAR	$0.5\times 0.7\times 1$	512$\times$512$\times$148–150	25/1.7	20	Philips 3T	46
ADAM	0.2–1 $\times$ 0.2-1 $\times$ 0.4–0.7	512-1024 $\times$ 512–1024 $\times$ 64–180	17.58–45.2 / 2.28–10.36	Multiple values	Philips 1, 1.5 or 3T	113

A total of 837 TOF-MRAs were collected from the aforementioned data sources as given in Table 1. Via manual inspection, we discarded 53 images due to poor image quality and finally, we left with 784 TOF-MRA images. We design experimenters to test the robustness of the proposed segmentation method in terms of the quantitative volumetric vessel segmentation performance along with its generalization capabilities across multi-site TOF-MRA datasets and preservation of the major vascular pathologies in the segmented volumetric representation of the vascular tree. Since UU-IAR and ADAM contain scans with pathologies, it is important to use samples from those two datasets in the test set. Also, the dataset is very diverse, with no set protocol used for all of the scans. So, it would be perfect to use the ADAM dataset as the test data to analyze the model’s multiple-site generalizability.

### Image annotation and dataset split

Manual voxel-wise vessel segmentation masks are publicly available for 54 subjects for the ITKTubeTK database. For the remaining five datasets, manual vessel segmentation masks are not provided. So, we follow a simple semi-automatic pipeline based on thresholding and region-growing followed by a manual voxel-wise correction to generate a voxel-wise vessel-segmentation mask. For the initial segmentation of the vascular tree, we have used the popular region growing-based algorithm called Grow-Cut^27^ implemented in 3DSlicer^28^. The foreground seed regions on the vessels were generated using adaptive Otsu’s thresholding and the background regions were marked manually. After the initial segmentation performed by the semi-automatic pipeline, manual voxel-wise correction of the segmentation results was performed by the junior raters from our group. The Junior raters are experienced in neuroimage segmentation and were only permitted to mark images individually until their performance reached the criteria of the gold standard by interacting with two expert radiologists from our research group. Using this semi-automatic pipeline we annotate the remaining 55 images from the ITKTubeTK dataset, and 50 images each from HH, IOP, and Guys. For UU-IAR and ADAM, all the images were manually annotated. For ADAM and UU-IAR manual segmentation masks for the pathologies viz. UIA and IAR are provided. Table 2 summarizes the datasets and dataset splits with different parameters.

**Table 2:** Demographics of the datasets used and data splits.

Dataset	# images (train/val/test)	#pathology (# images)	Annotation (vessel/pathology)
ITKTubeTK	109 (78/9/22)	0	Manual/na
HH	50 (45/5/0)	0	semi-automatic/na
IOP	50 (36/4/10)	0	Semi-automatic/na
Guys	50 (45/5/0)	0	Semi-automatic/na
UU-IAR	46 (23/23/0)	40 (36)	semi-automatic/manual
ADAM	0/0/113	129 (53)	semi-automatic/manual
Dataset summary			
Data split	# Samples	# Pathologies	# Scanner
Train	227	23	3
Val	46	23	3
Test	145	113	3

Dataset summary			
Data split	# Samples	# Pathologies	# Scanner
Train	227	23	3
Val	46	23	3
Test	145	113	3

### Experimental setup

Due to limited data and hardware resources, we pursued a patch-based training approach for our CNN models. We utilized a vessel centerline-based patch extraction strategy^3,4^ to create a training dataset with patches containing small vessels, as well as vessel crossovers and bifurcations for intermediate and large vessel structures^8,9^. We generated corresponding homotopic skeletonization and distance transform volumes from the ground truth volumes. During inferencing, non-overlapping patches covering the entire TOF-MRA volume were used (nnU-Net was applied with its default out-of-the-box configuration, automatically determining the patch size). We extracted 100 volumetric training patches of size $16\times 128\times 128$ from each TOF-MRA volume in the training set, resulting in a training dataset of 22, 700 patches that was sufficient to train all models without overfitting. TensorFlow:2.3 in Python was used to develop and train the CNN models, and experiments were conducted on the Google Cloud Platform with 32 vCPUs, 240 GB RAM, and two NVIDIA Tesla T4 GPUs.

### Experimental results

Six state-of-the-art deep learning-based 3D vessel segmentation methods, namely 3D U-Net^29^, Unception^6^, VC-Net^8,9^, BRAVE-NET^4^, nnU-Net^30^, and DeepVessel-Net^7^, are compared with the proposed method. Vesselness filters, parametric intensity-based segmentation methods, or 2D CNN are not being considered as they have already been proven inferior when compared with 3D vessel segmentation methods in the literature^4,8,9^. All models are trained with the same dataset split optimized with Adam (learning rate $10^{-4}$) until fully converged. Four evaluation metrics, namely Dice coefficient (Dice), Precision, and Average Surface Distance (ASD) implemented in MedPy (https://loli.github.io/medpy/), along with the Topological Coincidence (TC) between the ground truth or the voxel-wise label map *L* and the predicted segmentation $\hat{L}$ defined as,

1 $$\begin{aligned} TC = \frac{\sum _{\textbf{c}\in C} \varphi _{\hat{L}}(\textbf{c}) \cdot \delta _{L}(\textbf{c}) + \sum _{\textbf{c}\in C} \delta _{\hat{L} }(\textbf{c}) \cdot \varphi _{L}(\textbf{c}) +\epsilon }{\sum _{\textbf{c}\in C} \varphi _{\hat{L}}(\mathbf {\textbf{c}}) + \sum _{\textbf{c}\in C}\varphi _{L}(\textbf{c}) +\epsilon }, \end{aligned}$$

are used for quantitative evaluation and comparison. Here $C = \{\textbf{c}|\textbf{c}\in \mathbb {Z}^3\}$ represents a three-dimensional coordinate set and each coordinate triplet corresponds to a voxel. $\varphi _L$ denotes the homotopic skeletonization of *L* and $\delta _L$ represents morphological dilation of *L* (to reduce the impact of slight differences in vessel tracing).

**Figure 3:**
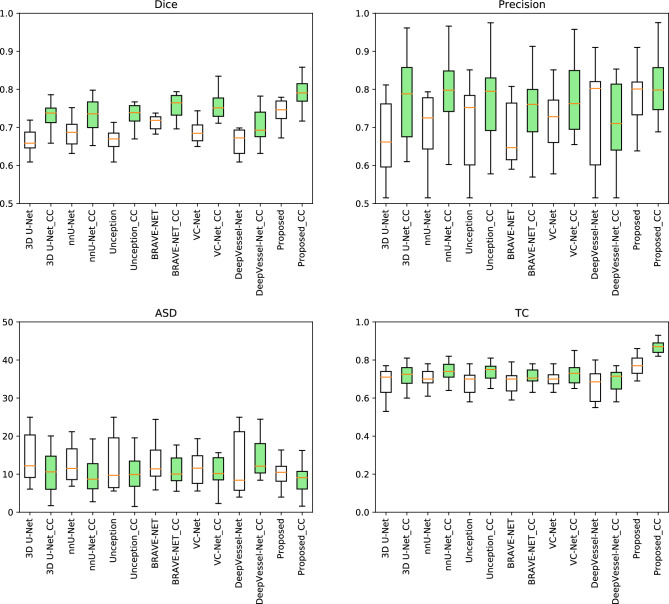
Quantitative performance of different models with (“Model_Name_CC”) and without the decorrelation loss on ADAM dataset.

**Figure 4:**
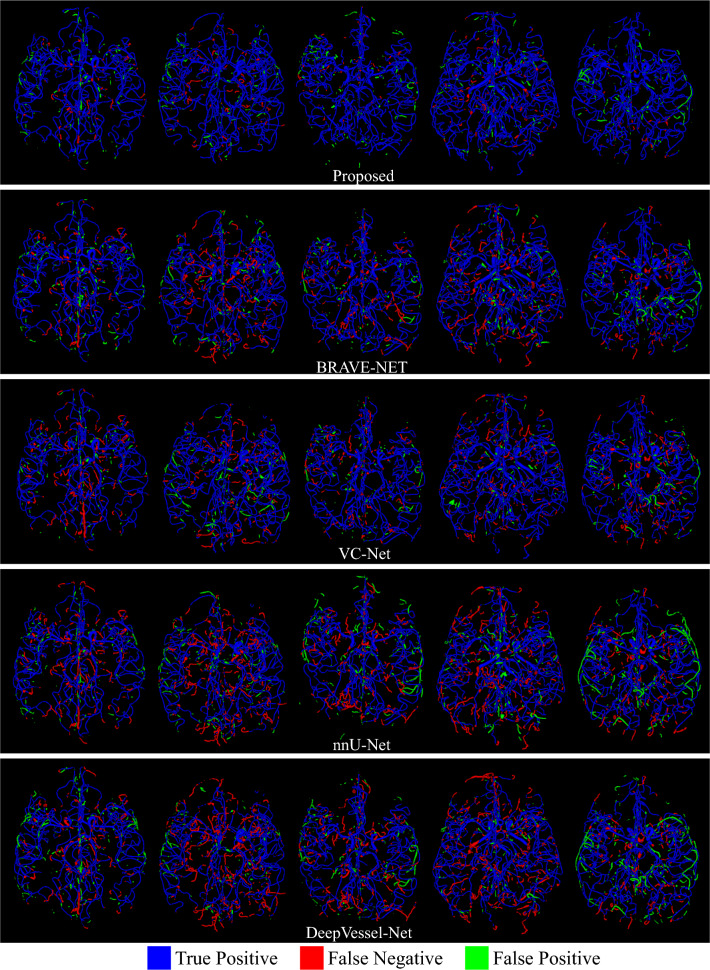
Qualitative segmentation results.

**Figure 5:**
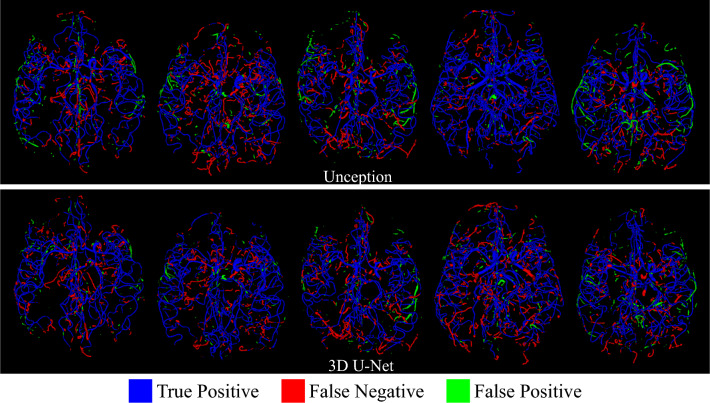
Qualitative segmentation results. True positive, false negative and false positive voxels are shown in blue, red, and green by comparing with the corresponding ground truth segmentation.

For a fair comparison with respect to the domain generalization capability of the compared and the proposed models, we train them with and without the decorrelation loss reported in Fig. 3 on the holdout test-set from the ADAM dataset (113 subjects). To better understand the segmentation performance of the proposed segmentation model we report the comparative performance in Table 3. Here we did not use the decorrelation loss during the model training as we are interested in the core segmentation performance of the models. Table 3 gives the mean and standard deviation of the segmentation scores of all the models on both the validation and holdout test sets. *p*-values of the statistical significance test regarding the Topological Coincidence (TC) between the proposed method and the six methods being compared are also reported in Table 3. Figures 4 and 5 depict the qualitative segmentation outcomes for five subjects, demonstrating a comparison between the proposed method and six state-of-the-art techniques *viz.* BRAVE-NET, VC-Net, nnU-Net, DeepVessel-Net, Unception, and 3D U-Net. True positive, false negative, and false positive voxels are shown in blue, red, and green by comparing with the corresponding ground truth segmentation. The visual analysis of these figures reveals that the proposed method exhibits a notable reduction in false negatives and false positives in comparison to the alternative methods, which makes it clinically more acceptable.

**Table 3:** Quantitative performance comparison of different models on both the validation and the hold-out test sets.

	Validation			Test			# Para. $\downarrow$	*p*-value
	Dice $\uparrow$	TC $\uparrow$	ASD $\downarrow$	Dice $\uparrow$	TC $\uparrow$	ASD $\downarrow$		
3D U-Net	0.71 ± 0.06	0.78 ± 0.14	1.65 ± 0.28	0.69 ± 0.02	0.75 ± 0.12	2.34 ± 0.31	$\sim$6 M	< 0.0001
Unception	0.72 ± 0.13	0.77 ± 0.11	1.63 ± 0.21	0.69 ± 0.01	0.71 ± 0.13	2.31 ± 0.35	$\sim$9 M	< 0.0001
VC-Net	$\mathbf {0.78 \pm 0.11}$	0.84 ± 0.13	$\mathbf {1.10 \pm 0.19}$	0.72 ± 0.02	0.76 ± 0.11	1.69 ± 0.45	$\sim$24 M	< 0.0001
BRAVE-NET	0.77 ± 0.08	0.82 ± 0.12	1.12 ± 0.21	0.71 ± 0.01	0.80 ± 0.09	1.72 ± 0.23	$\sim$10 M	< 0.001
nnU-Net	0.76 ± 0.11	0.81 ± 0.15	1.15 ± 0.23	0.71 ± 0.02	0.77 ± 0.10	1.42 ± 0.25	$\sim$6 M	< 0.0001
DeepVessel-Net	0.68 ± 0.12	0.75 ± 0.09	2.52 ± 0.35	0.65 ± 0.04	0.69 ± 0.18	2.39 ± 0.41	$\mathbf {\sim 0.06~ M}$	< 0.00001
Proposed	0.77 ± 0.10	$\mathbf {0.90 \pm 0.11}$	$\mathbf {1.10 \pm 0.15}$	$\mathbf {0.74 \pm 0.01}$	$\mathbf {0.87 \pm 0.01}$	$\mathbf {1.11 \pm 0.26}$	$\sim$8 M	–

### AI-assisted graphical user interface

**Figure 6:**
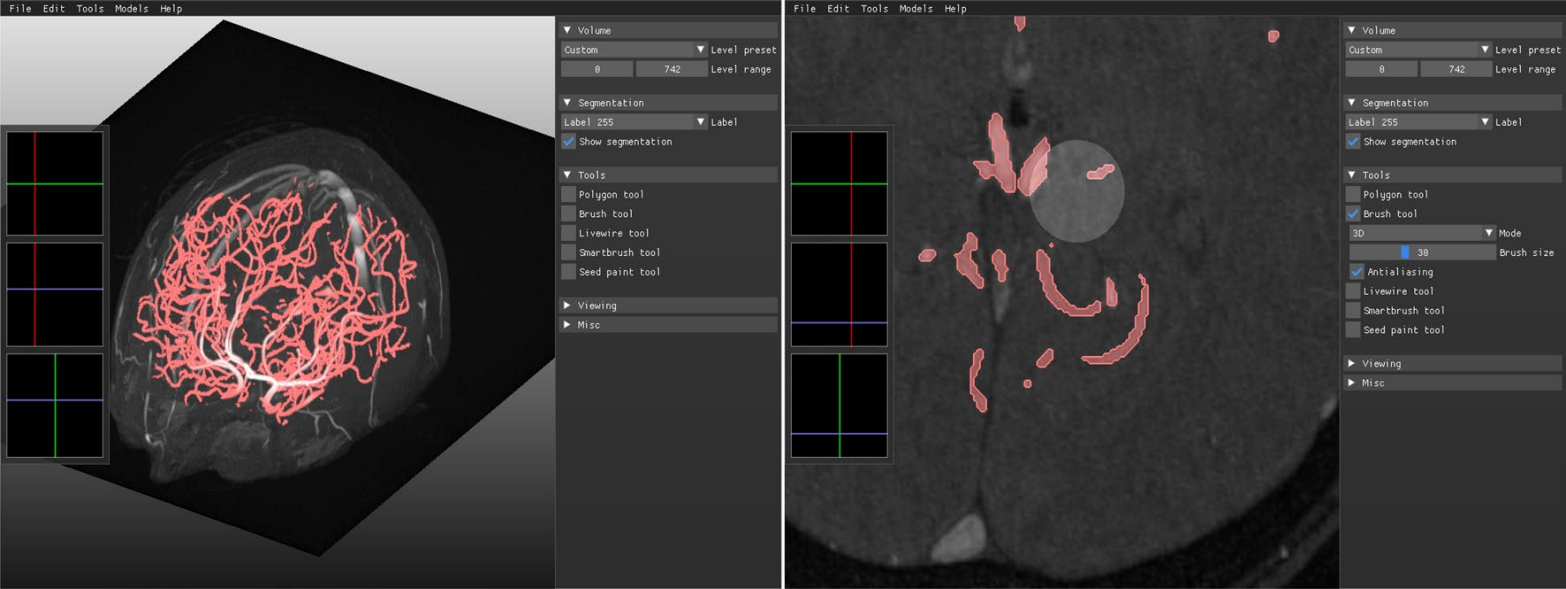
GUI for interactive editing of the automatic segmentation result of our proposed method.

We extend our GUI-based segmentation tool from https://github.com/FredrikNysjo/ichseg such that it can support interactive editing of the segmentation result of the proposed method. The GUI (see Fig. 6) is implemented in Python and OpenGL, can read DICOM data in addition to NIfTI and VTK volume files, and provides drawing tools for manual and semi-automatic segmentation and annotation. To extend the GUI to be able to apply trained models on loaded images, we store the required metadata (Anaconda environment name and other information) about each model in a single JSON file, which is read when the application is initialized. When the user selects a model from the GUI to generate an automatic segmentation, a separate process is launched and the corresponding Anaconda environment for the model is activated, after which the model is executed. Afterward, the generated segmentation mask is read back into the GUI for editing.

## Discussion

In this paper, we developed an AI-assisted clinical decision support system for the inspection of the intracranial cerebrovascular structure. To be a clinically feasible solution it should be robust and easy to use. The robustness of the developed system is studied in terms of its generalizability with respect to multi-center datasets. As evident from the experimental results, it can be observed that the proposed model achieved the best scores in all the qualitative performance measures. The proposed model beats its immediate competitor (BRAVE-NET) with around $2\%$ gain in the Dice score and around $6\%$ gain in the Topological Coincidence (Table 3). This means that the proposed method can preserve the topological structure along with very accurately segmenting the vascular structure (Fig. 3).

### Multi-center dataset generalization

**Figure 7:**
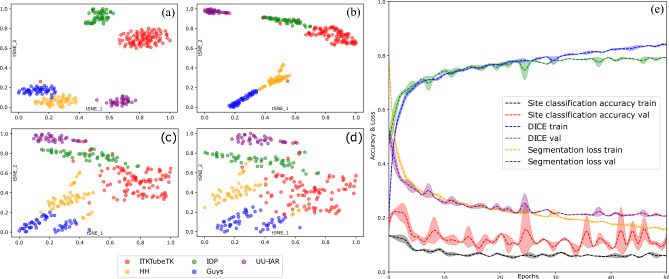
Latent features generated by the encoder network during the training process are plotted in 2D after applying tSNE (t-distributed stochastic neighbor embedding) (**a**) after the first epoch, (**b**–**d**) after 10, 30, and 50 epochs, (**e**) learning curves.

To better understand and demonstrate the effect of the decorrelation loss in the training process we present Fig. 7. This figure shows the learning curves for the model training and validation and model generalization during the supervised learning. As observed from Fig. 7a,e initially the MRA scans coming from the five different sites (ITKTubeTK, IOP, UU-IAR, HH, and Guys) form well-separated clusters. The model without the decorrelation loss learns how to segment the input images also encoding their source domain. Thus using the decorrelation loss we are able to remove scanner information during the course of the training process. This forces the model to learn how to segment the image while maximally reducing the domain bias as the training progress as observed by Fig. 7a–d. This is confirmed by the scanner classification accuracy being almost random chance after unlearning has been completed Fig. 7e. It can also be seen from the learning curves given in Fig. 7e that unlearning does not substantially decrease the performance on the main task i.e. vessel segmentation. The plot given in 7d can be considered as the best possible estimation of the performance of decorrelation loss as overfitting is observed after the 30th epoch.

### Pathology preserving vessel segmentation

**Figure 8:**
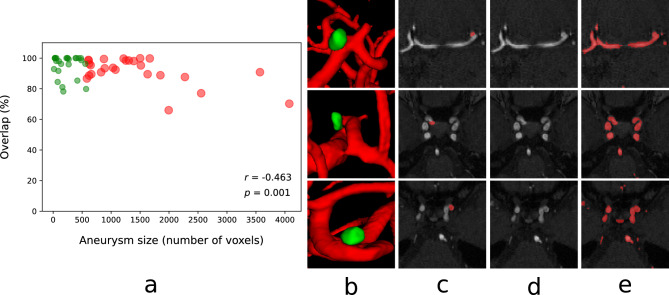
(**a**) Aneurysm volume vs its overlap percent with the segmented cerebrovascular structure generated by the proposed system. (**b**–**e**) Qualitative representation of aneurysms with the surrounding vessel structures in 3D and 2D views.

Another important aspect is how effectively the developed system can preserve the vascular pathologies in the 3D modeling of the segmented cerebrovascular structure. We further quantitatively analyze this by measuring the overlap percentage between the segmented pathology volume generated by expert radiologists and the vessel segmentation generated by the proposed AI-based system. We compute the percentage of the voxels correctly preserved in the segmented cerebrovascular structure for the ADAM dataset, where aneurysms of different sizes are present. Fig. 8a plots the aneurysm volume (number of voxels) and its overlap percent with the segmented cerebrovascular structure generated by the proposed system. Qualitative representation of the aneurysm with the surrounding vessel structures along with the 2D views are given in Fig. 8b–e. It can be observed from Fig. 8a that the proposed vessel segmentation model can preserve the different vascular pathological conditions very well. It achieves more than $80\%$ overlap in the case of small aneurysms which is even pretty hard for an expert radiologist to correctly detect from only the 2D slices.

## Methods

**Figure 9:**
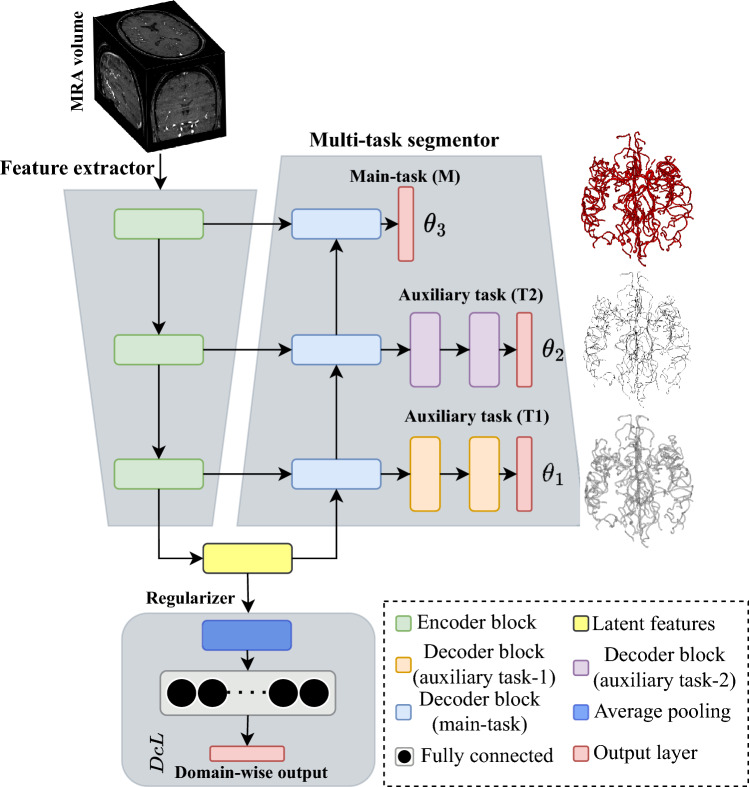
Model architecture.

The architecture of the multi-task deep CNN is illustrated in Fig. 9. It is composed of encoders and decoders, with a shared encoder and a partially shared main decoder. There are also exclusive decoding blocks for each of the related auxiliary tasks. Auxiliary tasks (*T*1 and *T*2) share some initial decoder blocks with the main task (*M*) but have their own decoders as well. Joint training, as proposed in^26^, utilizes shared decoders to aid the main decoder in learning intermediate representations and sharing important feature characteristics. Each encoder block consists of two 3D convolution layers with ReLU nonlinearity and one 3D MIP (Maximum Intensity Projection) layer that reduces the spatial dimension of the response map in half. Each decoder block of the main task contains one 3D RIP (Reverse Intensity Projection) layer, which uses the spatial location information from the corresponding encoder block to un-project the response map into twice the dimensions of the input along with two 3D convolution layers with ReLU nonlinearity. Residual and skip connections are employed within encoder and decoder blocks, as well as from the encoder to the decoder (main-task), to preserve small anatomical structures and ensure gradient flow. The network utilizes $3 \times 3 \times 3$ convolution kernels throughout and $2 \times 2 \times 2$ projection windows for the MIP and RIP layers.

To train three different tasks with distinct optimization objectives, three distinct loss functions viz. $\theta _1$, $\theta _2$, and $\theta _3$ are utilized. Let’s consider a 3D coordinate set $C = \{\textbf{c}|\textbf{c}\in \mathbb {Z}^3\}$, where each triplet of coordinates corresponds to a voxel. We define a 3D TOF-MRA image *X* and the corresponding voxel-wise label map *L* of dimensions $D \times W \times H$ such that $X:C\rightarrow \mathbb {R}$ and $L:C\rightarrow \{0, 1\}$. The values of *X* and *L* at position $\textbf{c}$ are represented by $X(\textbf{c})$ and $L(\textbf{c})$ respectively. The predictions for the two auxiliary tasks are denoted as $\widehat{J_1}$ and $\widehat{J_2}$, whereas the prediction for the main task is denoted as $\widehat{L}$. To calculate the loss $\theta _3$, the label map *L* is directly used. On the other hand, for computing loss $\theta _1$ and $\theta _2$, we generate the distance transform and skeleton maps from *L*. To compute the distance transform let us define the set of vessel voxels as $V = \{\textbf{v}|L(\textbf{v}) = 1\}$ and the set of vessel surface voxels as $S = \{\textbf{s}|L(\textbf{s})=1, \exists \textbf{u} \in \mathcal {N}(\textbf{s}), L(\textbf{u})=0\}$. Where $\mathcal {N}(\textbf{s})$ represent the 6-neighbourhood of voxel $\textbf{s}$, and let $\textbf{u}$ be a neighbourhood voxel with $L(\textbf{u})=0$. Then, for each vessel voxel $\textbf{v} \in V$ we can determine its distance transform value by calculating the distance from the nearest surface voxel as $\mathcal {D}(\textbf{v}) = \min _{\forall \textbf{s} \in S}||\textbf{v}-\textbf{s}||_2.$

The loss function $\theta _1$ is defined as the $smooth_{L1}$ loss, which is less affected by outliers and can prevent gradient explosions. This loss is expressed as, $\theta _1 = \sum _{\forall \mathbf {\textbf{v}} \in V}smooth_{L1}(\mathcal {D}(\textbf{v}) - \widehat{J_1}(\textbf{v}))$. Where $smooth_{L1}$ is defined as,

$$\begin{aligned} smooth_{L1}(z)= {\left\{ \begin{array}{ll} 0.5z^2/\beta &{} \text {if~} z<\beta \\ |z| - 0.5\beta &{} \text {otherwise}. \end{array}\right. } \end{aligned}$$

The Topological Coincidence (TC) between *L* and $\widehat{J_2}$ is quantified by the loss $\theta _2$ and can be defined as,

2 $$\begin{aligned} \theta _2 = 1- \frac{\sum _{\textbf{c}\in C} \widehat{J_2}(\textbf{c}) \cdot \delta _{L}(\textbf{c}) + \sum _{\textbf{c}\in C} \delta _{\widehat{L} }(\textbf{c}) \cdot \varphi _{L}(\textbf{c}) +\epsilon }{\sum _{\textbf{c}\in C} \widehat{T2}(\textbf{c}) + \sum _{\textbf{c}\in C}\varphi _{L}(\textbf{c}) +\epsilon }, \end{aligned}$$

Here $\varphi _Y$ refers to the homotopic skeletonization^31^ of *L*, while $\delta _L$ denotes morphological dilation of *L* to mitigate the effect of minor discrepancies in vessel tracking. It is worth noting that the computation of $\theta _2$ requires the prediction of both the primary task ($\widehat{L}$) and its own output ($\widehat{J_2}$), which serves as a form of regularization for the primary task. To optimize the primary task, we minimize the voxel-wise soft Dice loss^29^ between *L* and $\widehat{L}$ across all voxels, as follows,

3 $$\begin{aligned} \theta _3 = 1 - \frac{2\sum _{\textbf{c}\in C}L(\textbf{c})\cdot \widehat{L}(\textbf{c})+\epsilon }{\sum _{\textbf{c}\in C}L(\textbf{c}) + \sum _{\textbf{c}\in C}\widehat{L}(\textbf{c})+\epsilon }. \end{aligned}$$

The proposed model includes a regularization network as its third component, consisting of an average pooling layer, two fully connected layers, and a softmax layer. The network takes latent features from the encoder and produces category-wise predictions, which in this case correspond to the input’s domain prediction. During training, we observed that the model without the regularization network learned to segment the input images while encoding their source domain, leading to overfitting and a domain bias that resulted in decreased segmentation performance on data from unseen domains. To address this issue, we introduced an auxiliary loss term called Decorrelation Loss (DcL) to reduce the domain bias during training. The DcL minimizes the Pearson correlation coefficient between the actual and predicted domain labels, confusing the model about the dataset domains and forcing it to learn how to segment the image while minimizing the domain bias. For a given input MRA volume $x_i\in X$, the domain regularization network generates an output vector $P_i = {(p_{i1}, p_{i2}, \dots , p_{in}) : p_{ij}=[0,1]}$ representing the probability of the input MRA volume being in one of the *n* domains. The ground truth domain labeling is represented by $D_i ={(d_{i1}, d_{i2}, \dots , d_{in}) : d_{ij}=[0,1]}$ as a one-hot encoded vector. The Decorrelation Loss (DcL) is calculated as,

4 $$\begin{aligned} DcL = \frac{\sum _j(p_{ij}-\overline{p_{ij}})(d_{ij}-\overline{d_{ij}})}{\sqrt{\sum _j(p_{ij}-\overline{p_{ij}})^2(d_{ij}-\overline{d_{ij}})^2}}, \end{aligned}$$

where $\overline{p_{ij}}$ and $\overline{d_{ij}}$ represent the mean values of vectors $P_i$ and $D_i$ respectively.

“All methods were carried out in accordance with relevant guidelines and regulations and informed consent was obtained from all subjects and/or their legal guardian(s).”

## Data Availability

Five datasets viz. “ITKTubeTK” (https://public.kitware.com/Wiki/TubeTK/Data), “HH” (https://brain-development.org/ixi-dataset/), “Guys” (https://brain-development.org/ixi-dataset/), “IOP” (https://brain-development.org/ixi-dataset/), and ADAM (https://adam.isi.uu.nl/) are publicly available from the given sources. The current ethics approval of the dataset “UU-IAR” does not allow sharing of the original neuroimaging data.
